# Screening of Compounds against *Gardnerella vaginalis* Biofilms

**DOI:** 10.1371/journal.pone.0154086

**Published:** 2016-04-25

**Authors:** Cornelia Gottschick, Szymon P. Szafranski, Brigitte Kunze, Helena Sztajer, Clarissa Masur, Christoph Abels, Irene Wagner-Döbler

**Affiliations:** 1 Helmholtz Centre for Infection Research, Inhoffenstr. 7, 38124 Braunschweig, Germany; 2 Dr. August Wolff GmbH & Co. KG Arzneimittel, Sudbrackstrasse 56, 33611 Bielefeld, Germany; Fred Hutchinson Cancer Center, UNITED STATES

## Abstract

Bacterial vaginosis (BV) is a common infection in reproductive age woman and is characterized by dysbiosis of the healthy vaginal flora which is dominated by Lactobacilli, followed by growth of bacteria like *Gardnerella vaginalis*. The ability of *G*. *vaginalis* to form biofilms contributes to the high rates of recurrence that are typical for BV and which unfortunately make repeated antibiotic therapy inevitable. Here we developed a biofilm model for *G*. *vaginalis* and screened a large spectrum of compounds for their ability to prevent biofilm formation and to resolve an existing *G*. *vaginalis* biofilm. The antibiotics metronidazole and tobramycin were highly effective in preventing biofilm formation, but had no effect on an established biofilm. The application of the amphoteric tenside sodium cocoamphoacetate (SCAA) led to disintegration of existing biofilms, reducing biomass by 51% and viability by 61% and it was able to increase the effect of metronidazole by 40% (biomass) and 61% (viability). Our data show that attacking the biofilm and the bacterial cells by the combination of an amphoteric tenside with the antibiotic metronidazole might be a useful strategy against BV.

## Introduction

In nature, bacteria rarely live in suspensions, but are frequently attached to surfaces as biofilms. In such a way they seek protection in a community where sharing of nutrients, genetic exchange and protection, e.g. from antimicrobials, is ensured. This is true for bacteria living in ponds or water distribution systems as well as for bacteria residing in humans [1]. Since biofilms offer a stable mode of existence, biofilm forming bacteria can cause large health problems in the human body, e.g. when they are persisting in catheters and chronic wounds, develop on implants or are causative of chronic diseases, such as rhinosinusitis or osteomyelitis [2]. It can be extremely challenging to erase pathogenic biofilms that have formed on human tissues. Therefore strategies to attack them are diverse and include antibiotics alone or in combination with bioactive molecules or bacteriophages [3,4].

One of those challenges is bacterial vaginosis (BV), a vaginal infection which might be associated with biofilm formation and persistence with a prevalence of 10–50% in women worldwide [5–7]. It is characterized by a change in bacterial diversity from a uniform flora dominated by Lactobacilli such as *Lactobacillus crispatus*, *L*. *gasseri*, *L*. *iners* or *L*. *jensenii* to a flora that is highly diverse and mostly anaerobic [8,9]. Although its etiology is still unclear, *Gardnerella vaginalis*, but also *Atopobium vaginae*, *Prevotella sp*., *Sneathia sp*., *Mobiluncus sp*. and many others were frequently identified in women with BV [10]. BV causes malodorous vaginal discharge and can also lead to miscarriage, preterm birth and an increased risk of acquiring sexually transmitted infections such as HIV [11]. One of the criteria used for diagnosis (“Amsel” criteria) is the presence of at least 20% clue cells [12]. Clue cells can be identified microscopically and are vaginal epithelial cells covered with a layer of bacteria [13,14]. Although Amsel criteria were established already in 1983 it took more than 20 years until Swidsinski *et al*., using fluorescent in situ hybridization (FISH), were able to show that those clue cells are frequently covered by a biofilm consisting mainly of the facultative anaerobe *G*. *vaginalis* [15] which was only recently confirmed by Hardy et al. using peptide nucleic acid (PNA) probes [16]. There are now many culture based and non-culture based studies that identified *G*. *vaginalis* only as part of a multispecies biofilm [10,17,18]. However, some scientific reports have shown *G*. *vaginalis* as the big rascal of BV, since the majority of virulence factors it possesses is important for disease development. One publication directly tested *G*. *vaginalis* against the other bacterial vaginosis associated species in terms of adherence, biofilm formation and cytotoxicity and found that *G*. *vaginalis* had the strongest virulence potential [19]. But *G*. *vaginalis* also bears a great challenge. *In vivo* studies, for example, revealed that after successful therapy with oral metronidazole, which is currently the treatment of choice for BV, patches of biofilms consisting of *G*. *vaginalis* and *A*. *vaginae* persisted on epithelial cells [15,20]. The high rate of recurrence of up to 60% within 12 months of treatment may therefore be due to the lack of effectiveness of metronidazole against biofilms. Moreover, antibiotic treatment, especially when it occurs repeatedly, supports the development of resistant bacteria [21]. Therefore, developing strategies to destroy biofilms of *G*. *vaginalis* and possibly other biofilm associated bacteria might be a first step to develop a more sustainable way to treat BV and its recurrences. Various approaches concerning the effect of different substances on *G*. *vaginalis* have already been pursued: The antiseptic octenidine dihydrochloride was initially very effective against *G*. *vaginalis in vivo* but resulted in a high rate of resistance after a short period [22]. Another clinical trial showed that treatment with glycerol monolaurate kept *Lactobacillus species* intact and was able to inhibit growth of *G*. *vaginalis* [23]. The antimicrobial peptide Retrocyclin inhibited biofilm formation but not planktonic growth of *G*. *vaginalis* [24] and Thymol was able to inhibit formation of new *G*. *vaginalis* biofilms as well as destroy mature ones *in vitro* [25]. So far those substances have not been applied *in vivo* and therefore their efficiency has not been tested in women.

There are approaches with substances that are attacking the extracellular polymeric substance (EPS) that forms around biofilms, rather than attacking the bacteria, in order to make them susceptible to antibiotic treatment. DNase or the tenside lauramide arginine ethyl ester showed synergistic effects with antibiotics *in vitro* but have not been tested *in vivo* and are therefore not yet feasible for therapy [26,27]. A clinical study investigated whether boric acid, which is commonly administered against candidiasis, could disturb the biofilm in BV, but although the results looked promising after 2–3 months, the rate of recurrence after 38 weeks was unchanged [28]. An *in vitro* study showed that *G*. *vaginalis* can be displaced by *L*. *reuteri* and clinical trials that use different Lactobacillus species as probiotics in combination with antibiotics or alone showed potential [29,30].

BV is a multifactorial disease with a different flora and different problems in every affected woman. Therefore there is a need for medically applicable compounds that could be used either alone or in combination with antibiotics to treat BV and the physiological conditions which lead to BV recurrences. In our approach, as a first step, we analyzed different substances for their effectiveness against *G*. *vaginalis* biofilms. With the objective to use substances that have already proven successful in other antimicrobial settings, we tested four different categories of compounds on a *G*. *vaginalis* biofilm model of newly forming and already established biofilms, with the aim to identify new substances that could prevent, weaken or even destroy *G*. *vaginalis* biofilms. Those four categories were (1) antibiotics, (2) antibacterial enzymes and peptides, (3) antiseptics and (4) tensides.

Two antibiotics were tested: Tobramycin (TOB) is usually applied as treatment of *Pseudomonas aeruginosa* biofilms and has not yet been used against *G*. *vaginalis* biofilms. It blocks the bacterial protein synthesis. Metronidazole (MET) inhibits nucleic acid synthesis and is the current treatment of choice for BV. Its impact on *G*. *vaginalis* has already been investigated in previous studies either alone or in combination with additional substances [26,27,29,31].

We tested enzymes and peptides because extracellular proteins are important components of biofilms and they might help to degrade the biofilm matrix [32]. Two antibacterial hydrolases were selected: Lysozyme (LYS) that disrupts cell walls of bacteria and proteinase K (PRO) which degrades proteins [32,33]. Both are frequently used in the laboratory, but have not been applied as anti-biofilm treatments. As an antibacterial peptide, OP-145 (OP1) was tested. It interacts with membrane phospholipids and induces membrane thinning in bacteria and has been effective in the treatment of chronic middle ear infections [34] but was not tested against *G*. *vaginalis* biofilms before.

Antiseptics are commonly defined as substances that kill (bactericidal) or inhibit (bacteriostatic) the growth of bacteria [35]. The antimicrobial preservative chlorocresol (CLC, 4-chloro-3-methylphenol), the detergent cetylpyridinium chloride (CPC, 1-hexadecylpyridinium chloride) which can reduce gingivitis and was previously able to prevent dental plaque [36,37] and polyaminopropyl biguanide (PBI), also known as polyhexamethylene biguanide, that has been shown to be effective against *Staphylococcus aureus* amongst others and is used as disinfectant in swimming pools [38,39], were tested here because they have known antimicrobial properties and are already commercially used. Therefore obtaining approval for a new application would be simplified.

The fourth category of compounds studied here are surface-active agents (tensides) such as the emulsifier lecithin (LEC), which in combination with silver has been shown to be effective against biofilms on catheters due to its hydrophilic properties [40] and the amphoteric tenside sodium cocoamphoacetate (SCAA) that is frequently used in cosmetics and pharmaceutical products, but little has been published about it [41,42]. Due to their hydrophilic and hydrophobic moieties, amphoteric tensides have antibacterial properties. Their effectiveness against biofilms has not been studied yet.

## Material and Methods

### Strains and culture conditions

*Gardnerella vaginalis* strain ATCC 14018 was grown on Columbia agar plates (Becton Dickinson) with 5% sheep blood at 37°C in a 5% CO_2_ atmosphere. Liquid cultures were aerobically (5% CO_2_ atmosphere) grown in supplemented brain heart infusion medium (sBHI, Becton Dickinson) containing 2% (w/v) gelatin (Fluka), 0,5% yeast extract (Becton Dickinson), 0.1% starch (Merck) and 0.1% glucose (Sigma) for sBHIg Medium and 1% glucose (Sigma) for sBHIG medium, which was used to promote biofilm formation [25]. In one experiment (S3 Fig) *G*. *vaginalis* was inoculated in sBHI medium containing 0.1% maltose and for biofilm induction 1% maltose was used. The pH was adjusted to pH 7.0, pH 5.5, pH 5.0 or pH 4.5 using 1M HCl or 300 mM citrate-phosphate buffer according to the experimental design. Anaerobic experiments were performed in the Whitley MG1000 anaerobic workstation (dwscientific). Stock cultures were stored in sBHI containing 12.5% glycerin at -70°C. For some experiments, Todd Hewitt broth (THB, Becton Dickinson) and THB supplemented with 1% yeast extract (THBY, Becton Dickinson) were used.

### Compounds

**Fig 1:**
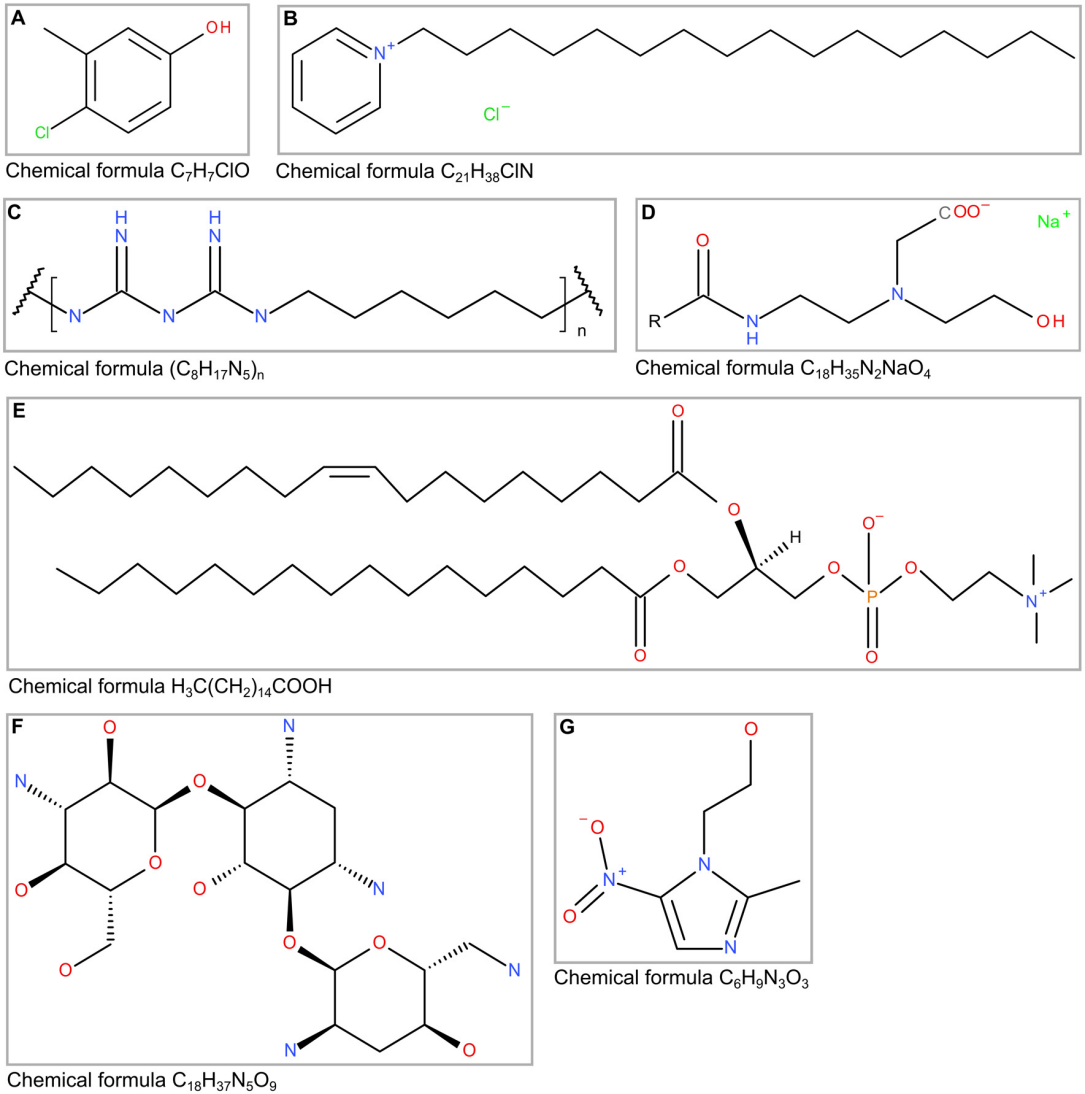
Chemical structures of tested compounds. CLC (A), CPC (B); PBI (C), SCAA (D), LEC (E), TOB (F) and MET (G).

The following compounds were tested: Chlorocresol (CLC, Clariant GmbH), Cetylpyridinium chloride (CPC, Fagron GmbH & Co. KG), Lecithin, (LEC, Cargill GmbH & Co. KG), Sodium cocoamphoacetate (SCAA, C.H. Erbslöh KG), Polyaminopropyl biguanide (PBI, Arch Biocides Ltd.), Lysozyme (LYS, Biozym GmbH), Metronidazole (MET, Biesterfeld Spezialchemie GmbH), OP-145 (OP1, OctoPlus N.V.), Tobramycin sulfate (TOB, Zhejiang Hisun Pharmaceutical Co. Ltd.), Proteinase K (PRO, Fermentas GmbH). All compounds, except LEC, which was dissolved in methanol (J.T. Baker), were dissolved or diluted in Milli-Q water and sterilized with 0.22 μm filters prior to usage (for abbreviations and concentrations see S1 Table). Compounds were diluted according to S1 Table and the correct pH of either pH 7 or pH 4.5 was again verified. The chemical structures of all antibiotics, antiseptics and tensides are shown in Fig 1.

### Planktonic cultures

An overnight culture of *G*. *vaginalis* cultured in sBHI was inoculated in media with different acidity (pH 4.5, pH 5, pH 5.5 or pH 7) at an optical start density of OD_600_ = 0.05, OD_600_ = 0.1 or OD_600_ = 0.2 according to the experimental design. The optical density (OD) was measured at 600nm over 25 hours.

### Biofilm cultures

A pre-culture was started from the *G*. *vaginalis* glycerol stock in sBHI and incubated over night at 37°C in a 5% CO_2_ atmosphere. This pre-culture was diluted to OD_600_ = 0.05 in sBHIG (pH 7.0) for the final biofilm culture. Similar to previously described experiments, for the biofilm formation prevention experiment, compounds were added at the beginning of the biofilm culture and analysis carried out after 20 hours of incubation. For the biofilm disruption experiment the medium was carefully removed after 20 h of incubation and fresh sBHIG medium containing the test compound was added to the existing biofilm and incubated for another 20 h under the same conditions [24,26]. sBHIG medium alone and the biofilm culture without compounds were applied as controls. Biofilms were then analysed after a total of 40 h incubation time. Biofilms were cultivated in Nunc^™^ MicroWell^™^ 96-Well Microplates (Thermo Scientific) for the crystal violet biofilm assay and Nunc^™^ MicroWell^™^ 96-Well Optical-Bottom Plates with Polymer Base (Thermo Scientific) for the biofilm viability assay.

### Analytical methods

Colony forming units (CFU) were determined from biofilms growing in 96-well microtitre plates (Thermo Scientific). Biofilms from triplicate wells were pooled and used for one CFU determination. After the appropriate incubation time, the biofilm was washed twice with phosphate buffered saline (PBS), scraped off and resuspended in 50 μl of 0.85% NaCl before serial dilution and spreading in triplicates onto Columbia agar plates with 5% sheep blood (Beckman). The plates were incubated at 37°C in a 5% CO_2_ atmosphere for 48 hours prior to CFU counting.

Biofilm mass was determined by crystal violet staining. The biofilm was grown as described and after the appropriate incubation time washed twice with PBS and dried for 15 min. The dried biofilm was then stained with 200 μl 2% crystal violet (Sigma) in ethanol per well and incubated at room temperature with shaking (15 min at 300 rpm). Then, the plate was washed with PBS three to five times until not specifically bound dye was removed. The plate was again dried for 15 min and subsequently crystal violet was extracted with 200 μl ethanol at room temperature over night at 300 rpm. 100 μl of the extracted solution were transferred to a new microtitre plate and absorbance was measured in a multi-label microplate reader (Wallac Victor 1420, Perkin Elmer life Sciences) at 620 nm. The percentage of biomass inhibition was calculated as *biomass inhibition* [%] = 100 –(100 * ((S-N)/(P-N))) where S is the average absorbance for the sample, N is the average absorbance for the negative control (medium) and P is the average absorbance for the positive control (biofilm without compound).

Biofilm viability was determined via live/dead staining using the live/dead BacLight Bacterial Viability Kit (Molecular Probes) according to the manufacturer’s protocol. Fluorescence was measured in a multi-label microplate reader (Wallac Victor 1420, Perkin Elmer life Sciences) at 530 nm excitation (green) and 630 nm excitation (red) and the percentage of viability inhibition was calculated as *viability inhibition* [%] = 100 –(100 * ((S^G^/S^R^)/(C^G^/C^R^))) where S^G^ is the average fluorescence at 530 nm and S^R^ is the average fluorescence at 630 nm for the sample and C^G^ and C^R^ are the respective values for the positive control (biofilm without compounds). For the negative control, the biofilm was killed by incubation with 70% 2-propanol for 5 minutes. For all conditions five or more replicates were prepared.

### Statistics

Results were plotted as mean ± standard deviation of the mean from at least triplicates. The error of the viability inhibition was calculated by the formula (1/B^2^)*(√(B^2^*a^2^+A^2^*b^2^))*100 where A is the mean of the compound treated sample, a is the standard deviation of the compound treated sample, B is the mean of the untreated positive control and b is the standard deviation of the untreated positive control.

## Results

### *G*. *vaginalis* biofilm model

**Fig 2:**
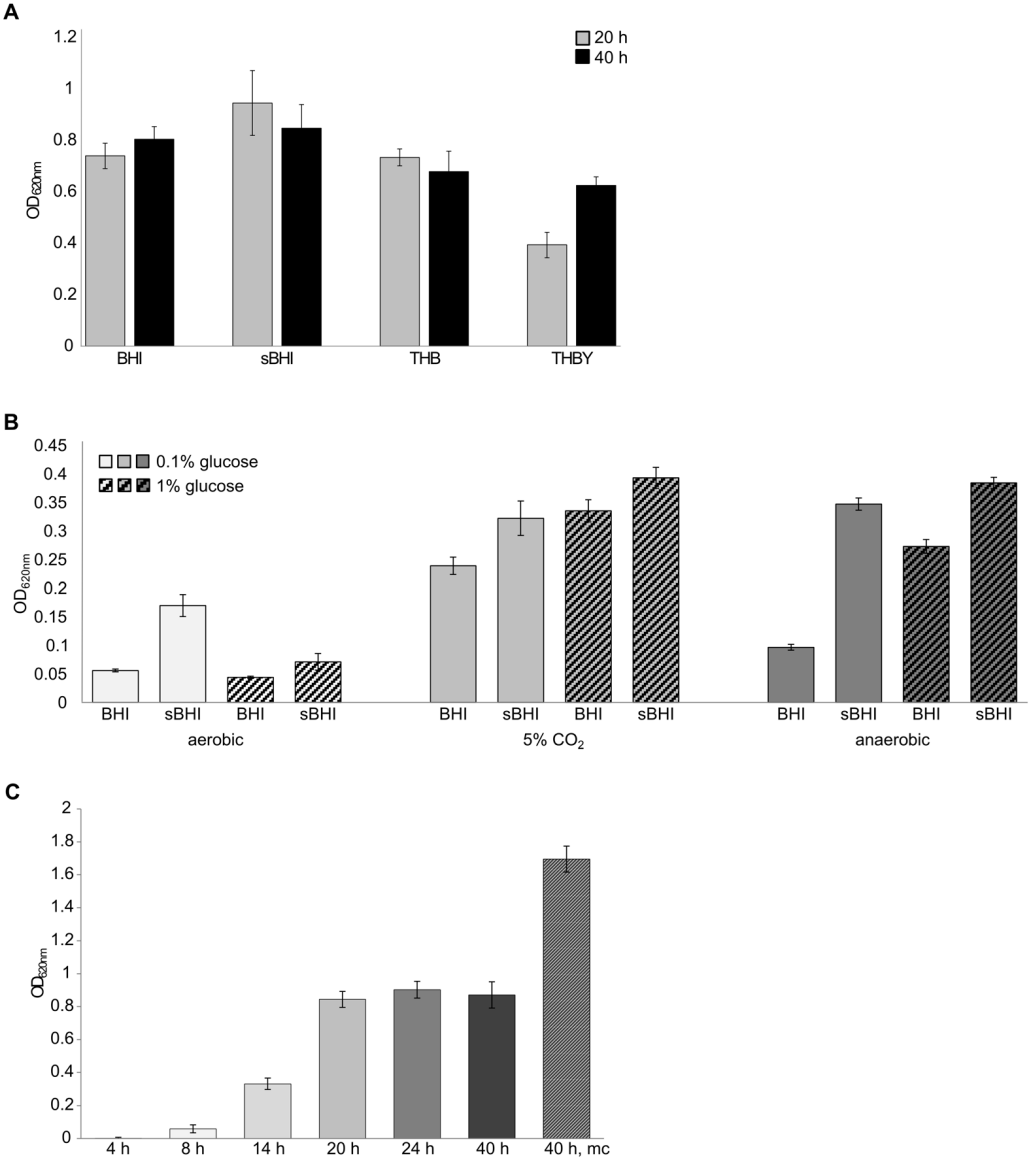
Effect of medium and culture conditions on the biomass of *G*. *vaginalis* biofilms. (A) Biomass development in different media after 20 h and 40 h of growth. (B) Effect of glucose concentration and oxygen. Biofilm mass was determined by crystal violet staining. Mean and standard deviation from twelve replicate cultures are shown. (C) Aerobic growth (5% CO_2_) at 37°C in sBHIG was measured by crystal violet staining. For the 40 h mc value, the growth medium had been replaced by fresh medium after 20 h of incubation (mc = medium change).

In order to develop a biofilm model of *G*. *vaginalis*, first, the media BHI, sBHI, THB and THBY were tested under aerobic conditions in a 5% CO_2_ atmosphere and biofilms were grown for either 20 h or 40 h. The best biofilm formation could be observed for sBHI medium followed by BHI (Fig 2A). Then, the effect of glucose supplementation and oxygen were investigated for the media BHI and sBHI with 0.1% or 1% glucose respectively in a 5% CO_2_ atmosphere or under anaerobic conditions. Biofilm formation was strongest in the 5% CO_2_ atmosphere, followed by the anaerobic condition, and was weak under aerobic conditions. Glucose enhanced biofilm formation slightly, but resulted in even less biofilm formation under aerobic conditions. The strongest biofilm formation was obtained on sBHI supplemented with 1% glucose (sBHIG) in the 5% CO_2_ atmosphere (Fig 2B). Therefore further biofilm experiments were performed under these conditions.

Biofilm formation was observed until 20 h of incubation; afterwards a plateau was reached representing an established biofilm. Replacing the spent medium by fresh medium at 20 h (medium change = mc) resulted in further biofilm formation and biofilm mass was doubled at 40 h compared to 20 h (Fig 2C).

**Fig 3:**
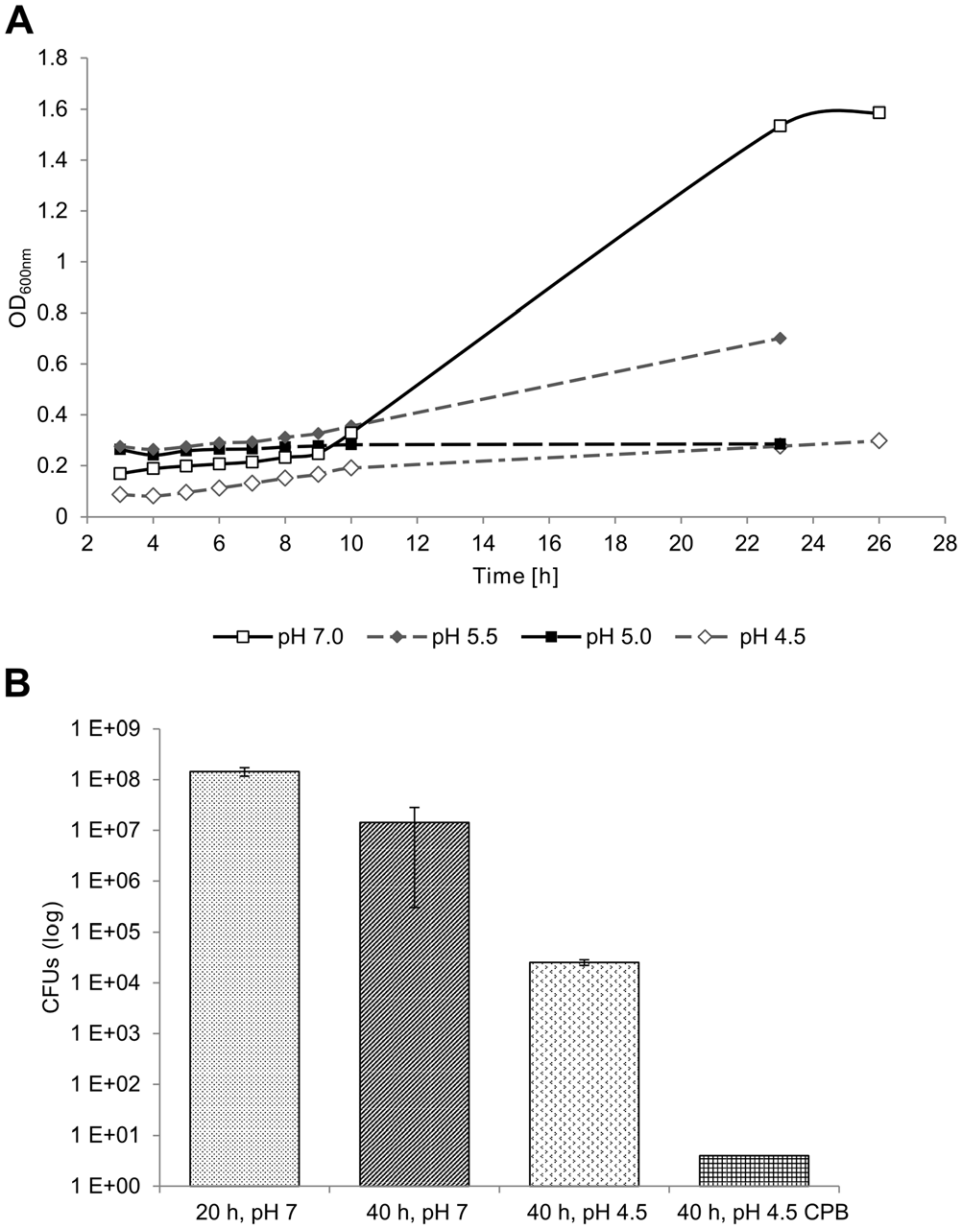
Effect of pH on growth of *G*. *vaginalis* in planktonic and biofilm culture. (A) Planktonic growth in media adjusted to pH 7, 5.5, 5 or 4.5 measured via OD_600nm_ (B) Biofilm formation after 20 h at pH 7 (20 h, pH 7) and after 40 h with a medium change after 20 h. After the medium change, the pH was either kept at pH 7 (40 h, pH 7), changed to pH 4.5 (40 h, pH 4.5) or buffered with citrate phosphate buffer (CPB) to pH 4.5 (40 h, pH 4.5 CPB). Biofilm formation was measured using colony forming units (CFUs). Mean and standard deviation from triplicate cultures are shown.

We then tested the effect of pH on biofilm formation. BV is characterized by a shift of the pH from 4.5 to neutral or alkaline conditions. *G*. *vaginalis* prefers to grow under neutral conditions (Fig 3A). Therefore we tested whether a shift to an acidic pH could disrupt an established biofilm. For this experiment, a *G*. *vaginalis* biofilm was grown for 20 h in unbuffered medium where the pH had initially been adjusted to pH 7. We then replaced the medium by (1) unbuffered medium adjusted to pH 7, (2) unbuffered medium adjusted to pH 4.5, or (3) a medium buffered to pH 4.5 using citrate phosphate buffer (CPB). The biofilm was then allowed to grow for additional 20 h and then CFUs were determined. Fig 3B shows that shifting the pH to 4.5 after 20 h of biofilm formation reduced CFU counts by three orders of magnitude, and when the pH was stabilized at pH 4.5 by a buffer, the established biofilm was almost completely disintegrated; less than 10 CFUs could be detected. PH did not only affect the ability to form CFUs, but also affected the biofilm mass (S1 Fig). Therefore, a low pH alone strongly influences biofilm formation and integrity of *G*. *vaginalis* biofilms even when the biofilm had already established. For measuring the effect of various compounds on *G*. *vaginalis* biofilm formation we used an unbuffered neutral medium in order to mimic the situation in BV *in vivo*.

We conducted two types of experiments: (1) In the biofilm formation experiment, test compounds were added at the beginning of incubation to determine their effect on newly forming biofilms. Measurements took place after 20 h of biofilm formation. (2) In the biofilm prevention experiment, biofilms were grown for 20 h without the test compounds, and then the spent medium was replaced by fresh medium containing the test compounds to determine if existing biofilms could be destroyed. Measurements were performed after 40 h of biofilm formation. Biomass was measured with crystal violet staining and viability was measured using live/dead staining. Positive and negative controls of the viability assay were verified once in the beginning (S2 Fig).

### Effect of antibiotics on the *G*. *vaginalis* biofilm

**Fig 4:**
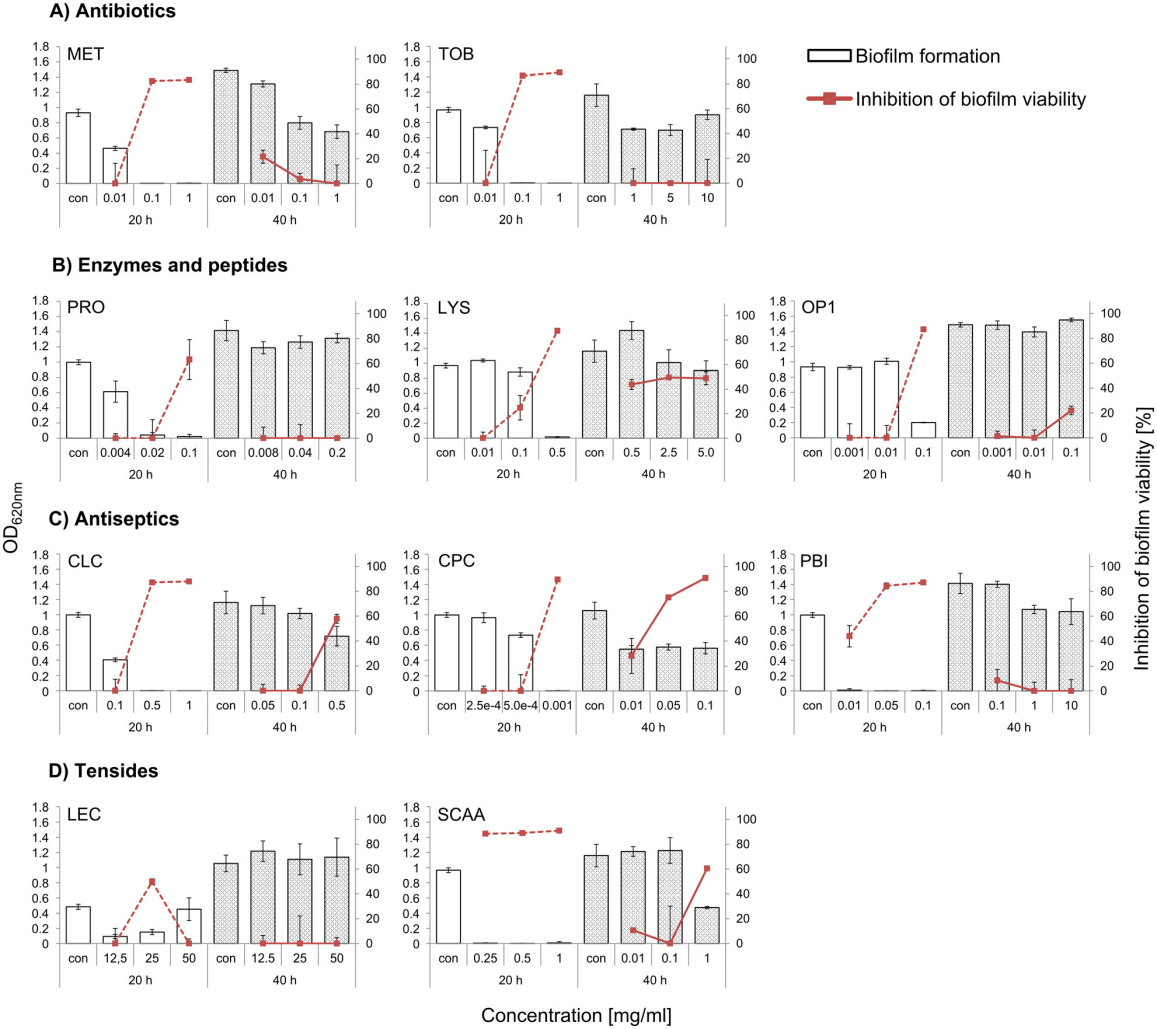
Effect of test compounds on *G*. *vaginalis* biofilm mass and viability. (A) antibiotics, (B) enzymes and peptides, (C) antiseptics and (D) tensides. Biofilm mass was determined by CV staining and is shown on the primary y-axis. Inhibition of biofilm viability [%] was measured via live/dead staining and is shown on the secondary y-axis. Mean and standard deviation from triplicate cultures are shown.

Two antibiotics were tested (Fig 4A). As expected, MET was able to prevent the development of *G*. *vaginalis* biofilms as reflected by very little biomass and a large inhibition of viability in the biofilm formation experiment. Interestingly, application of TOB to *G*. *vaginalis* biofilms showed a very similar result to that of MET and was able to inhibit biofilm formation at the same concentration as that used for MET. When MET was added after 20 h of biofilm formation, further biofilm growth was prevented, but the existing biofilm could not be disintegrated and the viability remained fully intact after a total of 40 h using a concentration of 0.1 mg/ml and higher. Again, the same was observed for TOB although even higher concentrations were tested. Therefore, as long as bacteria were growing and metabolically active, both antibiotics were very effective, but neither of them was able to disintegrate an existing biofilm, which is why further compounds were tested.

### Effect of antimicrobial enzymes and peptides on the *G*. *vaginalis* biofilm

Two enzymes and one peptide were tested (Fig 4B). The peptide OP1 was among the compounds with the weakest effect. Although biofilm viability was highly impaired, it still allowed biofilm formation in the biofilm formation experiment and did not show any effect in the biofilm disruption experiment. PRO and LYS on the other hand were able to prevent biofilm formation completely and also inhibited biofilm viability strongly (after 20 h). In the 40 h experiment, however, only LYS was able to prevent further biofilm formation. It could not resolve the existing biomass, but was able to affect 50% of the biofilm viability (unlike the antibiotics). By contrast, in the biofilm disruption experiment, PRO had a small effect on biofilm mass and none on its viability (Fig 4B). In this category, LYS which degrades the bacterial cell wall, was the most potent compound because it prevented new biofilm formation and disturbed the established biofilm.

### Effect of antiseptics on the *G*. *vaginalis* biofilm

Since it would be challenging to administer LYS without side effects, further substances that have already been applied for other purposes were tested. All antiseptics (Fig 4C) provided satisfying results in the biofilm formation prevention experiment. They prevented biofilm formation and inhibited viability to a very high extent. Differences between the compounds could be observed in the biofilm disruption experiment. Here, CPC was able to not only prevent further biofilm formation, but because the mass of the treated biofilm was less than the biofilm mass of the control in the 20 h experiment, it was able to even disintegrate the established biofilm. Accordingly, viability was inhibited by almost 100%. Of the antiseptics, CLC was the compound with the second best result after 40 h. Similar to CPC, it not only prevented further biofilm formation, but was also able to reduce the existing biofilm mass and showed around 60% of viability inhibition. PBI on the other hand, did not affect biofilm viability and was not able to reduce the established biofilm. Therefore, of all the antiseptics CPC showed the best result and was effective at the lowest concentrations (0.001 mg/ml in the 20 h experiment and 0.1 mg/ml in the 40 h experiment).

### Effect of tensides on the *G*. *vaginalis* biofilm

We tested two tensides, SCAA and LEC (Fig 4D). Whereas LEC had little effect in preventing biofilm formation and none in disrupting biofilms, SCAA was highly effective. In the 20 h experiment it prevented the formation of *G*. *vaginalis* biofilm completely. In the 40 h experiment, it dissolved more than 50% of the established biofilm and inhibited 60% of biofilm viability. It is therefore the most potent biofilm dissolver of this study, followed by CRC although higher concentrations of SCAA were needed (0.25 mg/ml in the 20 h experiment and 1 mg/ml in the 40 h experiment).

**Fig 5:**
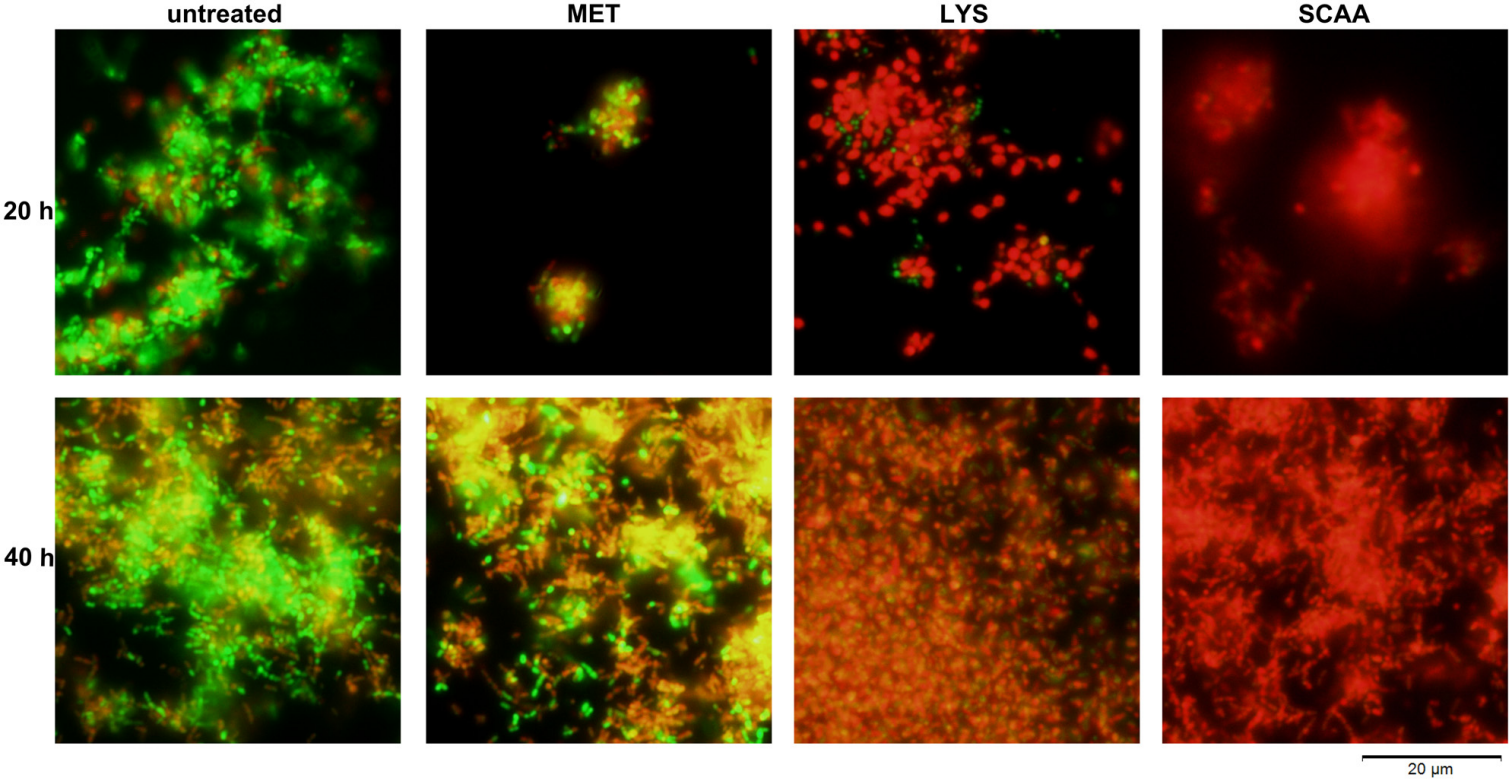
Viability of *G*. *vaginalis* biofilms after treatment with an antibiotic, enzyme or tenside. Live/dead staining of *G*. *vaginalis* biofilms treated with 0.1 mg/ml MET, 0.5 mg/ml LYS or 1 mg/ml SCAA is shown for the 20 h experiment and the 40 h experiment in comparison to the untreated control.

Fig 5 shows the viability of *G*. *vaginalis* biofilms treated with MET, LYS or SCAA. For these pictures, biofilms were scraped off the microtiter plate bottoms, therefore cell densities are not representative of the biomass. In the untreated biofilms, viability was very high throughout the experiment, i.e. after 20 h of growth and after 40 h of growth. After MET treatment (0.1 mg/ml), cell viability was weakly reduced. Many cells in the MET treated biofilm were still viable, and those bacteria, which have not been killed by MET, can probably regrow the biofilm and might be responsible for recurrence of BV. Biofilms treated with LYS (0.5 mg/ml) showed membrane damage of almost all cells. Because LYS degrades the cell walls, those bacteria cannot resist the osmotic pressure in the medium and increase in volume, especially in the biofilms of the biofilm formation prevention experiment. SCAA treated biofilms (1 mg/ml) consisted of red fluorescing dead cells only. A very strong inhibition of biofilm viability of newly forming and established biofilms could be observed with this compound.

Since it was previously shown that the carbon substrate can influence biofilm formation, we tested the effect of MET (0.1 mg/ml), LYS (0.5 mg/ml) and SCAA (1 mg/ml) on biofilms induced by maltose instead of glucose [43]. Unlike for biofilms that used glucose as carbon substrate, none of the compounds was able to prevent biofilm formation completely after 20 h. Nevertheless, they were all able to reduce its biomass and inhibited biofilm viability strongly with SCAA again as the most effective compound. Also the effect in biofilm prevention was strongest with SCAA treatment and showed a strong reduction in biofilm mass and viability to a similar extent than when biofilms were grown with glucose (S4 Fig). Overall, biofilm mass and viability were slightly less affected when maltose was used as carbon substrate, but compounds were still able to disturb the biofilm. Therefore the biofilm damaging effects of the compounds analysed here is not strongly dependent on the carbohydrate source.

### Effect of SCAA in combination with other compounds on the *G*. *vaginalis* biofilm

**Fig 6:**
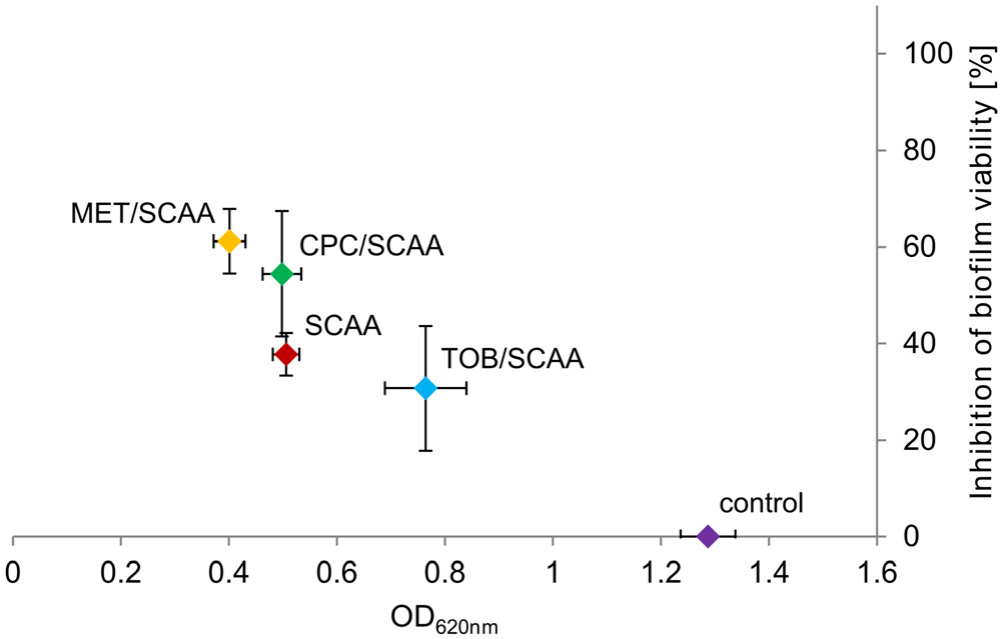
Biofilm mass and viability after treatment with combinations of tensides and antibiotics. Biofilm mass was determined by crystal violet staining, and inhibition of biofilm viability was determined by live/dead staining. The tenside SCAA (1 mg/ml) was combined with 25 mg/ml TOB, 0.1 mg/ml MET, 0.05 mg/ml CPC or applied alone and compared to the untreated control. Mean and standard deviation from triplicate cultures are shown.

Since SCAA was a very promising compound it was subsequently tested in combination with other compounds. We evaluated the effect of SCAA (1 mg/ml) on established *G*. *vaginalis* biofilms (biofilm prevention experiment) in combination with antibiotics (MET, 0.1 mg/ml or TOB, 25 mg/ml) and in combination with the very effective antiseptic CPC at the lowest effective concentration determined above (0.05 mg/ml). Surprisingly, Fig 6 shows that the effect of SCAA on biofilm mass was weakened in combination with TOB, and its inhibition of viability was only weakly increased in combination with CPC. However, inhibition of both biofilm mass and viability was increased in combination with MET. It was only weakly increased compared to SCAA alone, but could increase the effectiveness of MET alone by 41% (biomass) and 60% (viability) (Fig 4A). Furthermore, other amphoteric substances with different fatty acid chain lengths than SCAA were tested but showed no enhanced effect alone or in combination (S3A and S3B Fig). Therefore, SCAA and MET was the best anti-biofilm combination identified in this study.

## Discussion

In recent years, many studies have pointed out the importance of *G*. *vaginalis* in BV. Using high throughput sequencing of the 16S rRNA gene, it could be shown that it is frequently present in women with BV and although healthy women can also be colonized by *G*. *vaginalis*, its properties are of higher virulence in diseased women [8,44–49]. Therefore, testing substances on a *G*. *vaginalis* biofilm is a first, simplified, but valid approach to find new strategies against BV.

In order to develop this *G*. *vaginalis* biofilm model, different media and growth conditions were tested. In accordance with published biofilm models, sBHI supplemented with glucose was the medium for which the best growth of *G*. *vaginalis* was observed. We could show that the addition of 1% glucose resulted in better biofilm formation than 0.1% glucose. Other biofilm models used 0.3% glucose [24,26] and we therefore suggest using a higher concentration of glucose or switch to maltose as proposed by Machado et al. [43]. They showed that maltose induced a stronger biofilm growth of their strains. Therefore we tested our compounds in the concentrations that proved effective on the glucose induced biofilm model on a maltose induced biofilm. We could show that the compounds were very effective in disturbing the newly developing or established biofilm albeit by a weaker extent than we tested before on the glucose induced biofilm. This is most likely due to the higher biomass of the maltose induced biofilm and higher concentrations of our substances would probably lead to the same results we have shown for glucose as biofilm inducer.

We were able to show a strong effect of the pH on *G*. *vaginalis* biofilm formation. This is in accordance with the observation that during BV the pH increases, providing suitable conditions for the growth of various species including *G*. *vaginalis* [50]. Thus, lowering of the pH of the vaginal milieu, e.g. by using lactic acid containing pessaries, might be sufficient to suppress *G*. *vaginalis* and thereby avoid BV recurrences [51]. However, acid treatments may only give transient relief and BV recurrences might again emerge after acidification of the vagina with such products is terminated. We therefore strive to find a cure that kills *G*. *vaginalis* in its preferable conditions which are the conditions during BV in order to get rid of a *G*. *vaginalis* biofilm in the long run.

We tested four different categories of substances. Both antibiotics were highly effective against growing cells of *G*. *vaginalis*, but could not harm the established biofilm which is concurrent with the fact that planktonic growth of *G*. *vaginalis* can be inhibited by metronidazole at the same concentration that we found effective in our study [52]. The best results were obtained with the antiseptic CPC and the amphoteric tenside SCAA. Both not only prevented formation of a new biofilm but also disintegrated the mature biofilm and strongly inhibited its viability. In combination, they did not benefit from each other. However, even if CPC had been the most effective compound identified in this study, it would not be wise to apply it to mucosal surfaces such as the vaginal epithelium because it could lead to irritations and it has not been studied what CPC does to commensal Lactobacillus species. SCAA on the other hand is a tenside that is frequently used as an ingredient for cosmetics and based on the chemical structure and its properties is thought to be safe for human use. Therefore, a clinical study (ClinicalTrials.gov identifier: NCT02687789) has been implemented in order to analyse whether amphoteric tensides such as SCAA are applicable to mucosal surfaces and do not irritate the vaginal epithelium.

For eradication of a persisting biofilm, the extracellular polymeric substance (EPS) around it has to be considered. This EPS matrix contains polysaccharides, proteins, DNA and lipids and can buffer or eliminate the effect of bioactive molecules like antibiotics. Moreover, some cells within a biofilm shift to a metabolically inactive state and thus common molecular targets of antibiotics, e.g. cell wall synthesis, protein synthesis, and RNA or DNA synthesis, are weakly expressed. For those reasons, bacteria growing in biofilms can be hard to eliminate [1]. They may be partly deactivated by antibiotic treatment but are surviving in small patches which can be reactivated over time [53,54]. It would be interesting to test if the tenside SCAA disintegrates the EPS, thereby breaking up the biofilm and activating bacterial metabolism, such that the antibiotic metronidazole can kill the cells and at the same time keep commensal Lactobacillus species intact [27]. Since SCAA showed increased effectiveness in combination with metronidazole, it could be of great value in therapy where they can be applied simultaneously. Of the existing *in vitro* studies which found compounds against *G*. *vaginalis*, only three investigated substances that acted on biofilms and only one substance specifically attacked the EPS. Compounds that have been found to be effective against the biofilm were retrocyclin, thymol, subtilosin and lauramide arginine ester which were all effective in lower concentrations than what we propose to use for SCAA (1 mg/ml) [24,25,27]. DNase specifically targeted the EPS and was also effective at lower concentrations [26]. Therefore degrading the extracellular DNA of the biofilm matrix with DNase could be a potent approach, which has to be pursued concerning its safety and efficacy. On the basis of our data we suggest to use a relatively high concentration of SCAA which exhibits a rather low irritation potential as compared to other tensides. Verifying these results with multiple G. vaginalis isolates or the introduction of a multispecies biofilm model that includes commensal as well as pathogenic bacteria would be additional ways to further investigate the effects of amphoteric substances like SCAA. Here, an ongoing clinical study in humans (ClinicalTrials.gov identifier: NCT02687789) will evaluate whether amphoteric tensides such as SCAA are safe and promising additional substances to prevent or even treat recurrent BV by targeting biofilms.

## Supporting Information

S1 Fig pH effect on the biomass of *G*. *vaginalis* biofilms. Biofilm formation after 20 h at pH 7 (20 h, pH7) and after 40 h with a medium change after 20 h. After the medium change, the pH was either kept at pH 7 (40 h, pH 7), changed to pH 4.5 (40 h, pH 4.5) or buffered with citrate phosphate buffer (CPB) to pH 4.5 (40 h, pH4.5 CPB). Biofilm formation was quantified via crystal violet stain. Mean and standard deviation from triplicate cultures are shown.
(TIF)

S2 Fig Positive and negative control of the viability assay. For the positive control, live/dead staining was performed with an untreated 20 h or 40 h old biofilm. The same biofilms were then killed with 70% 2-propanol for the negative control.
(TIF)

S3 Fig Effect of active compounds on maltose induced biofilms. Maltose induced *G*. *vaginalis* biofilms were challenged with MET (0.1 mg/ml), LYS (0.5 mg/ml) and SCAA (1 mg/ml). Biofilm mass was determined by CV staining and is shown on the primary y-axis. Inhibition of biofilm viability [%] was measured via live/dead staining and is shown on the secondary y-axis. Mean and standard deviations from five replicates are shown.
(TIF)

S4 Fig Effect of tensides on *G*. *vaginalis* biofilms. (A) C1, Cocoamidopropyl hydroxysultaine; C2, disodium cocoamphodiacetate; C3, sodium cocoamphopropionate; C4, sodium lauroamphoacetate; C5, cocoamidopropyl betaine (SCAA). (B) Effect of those tensides in different combinations with each other and with SCAA compared to C4 and SCAA alone. The primary y-axis shows biofilm mass determined by crystal violet staining, and the secondary y-axis shows inhibition of biofilm viability determined by live/dead staining. Mean and standard deviation from six replicate cultures are shown.
(TIF)

S1 Table Summary of all tested compounds including abbreviations, providers, tested concentrations and numeric values. (PDF)
