# Supplementary figures and images for: Screening of Compounds against Gardnerella vaginalis Biofilms
Source: PLoS One. 2016 Apr 25;11(4):e0154086. doi: 10.1371/journal.pone.0154086 (PMC4844189; doi:10.1371/journal.pone.0154086)

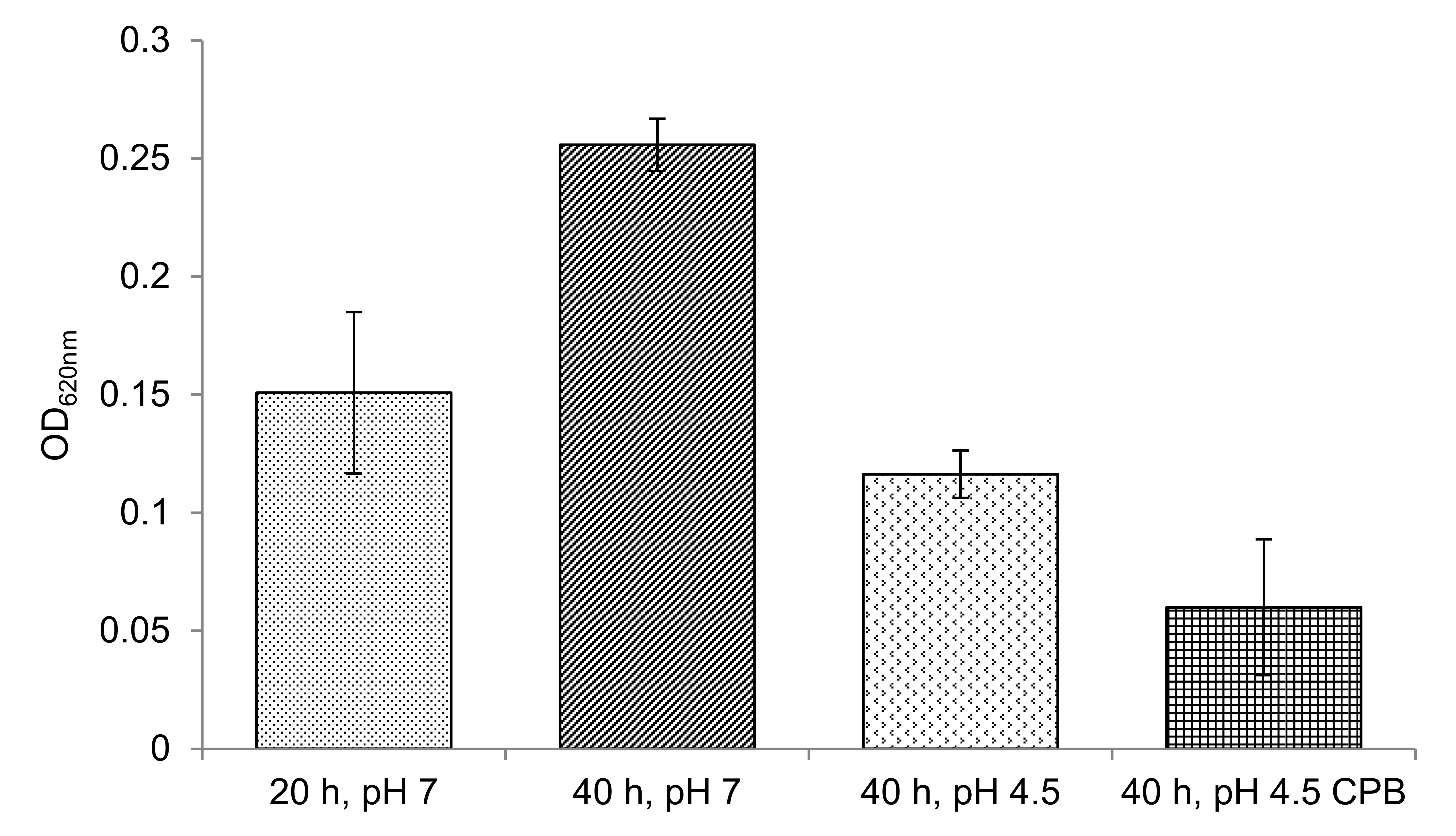

Supplement: S1 Fig — Biofilm formation after 20 h at pH 7 (20 h, pH7) and after 40 h with a medium change after 20 h. After the medium change, the pH was either kept at pH 7 (40 h, pH 7), changed to pH 4.5 (40 h, pH 4.5) or buffered with citrate phosphate buffer (CPB) to pH 4.5 (40 h, pH4.5 CPB). Biofilm formation was quantified via crystal violet stain. Mean and standard deviation from triplicate cultures are shown. (TIF) [file pone.0154086.s001.tif]

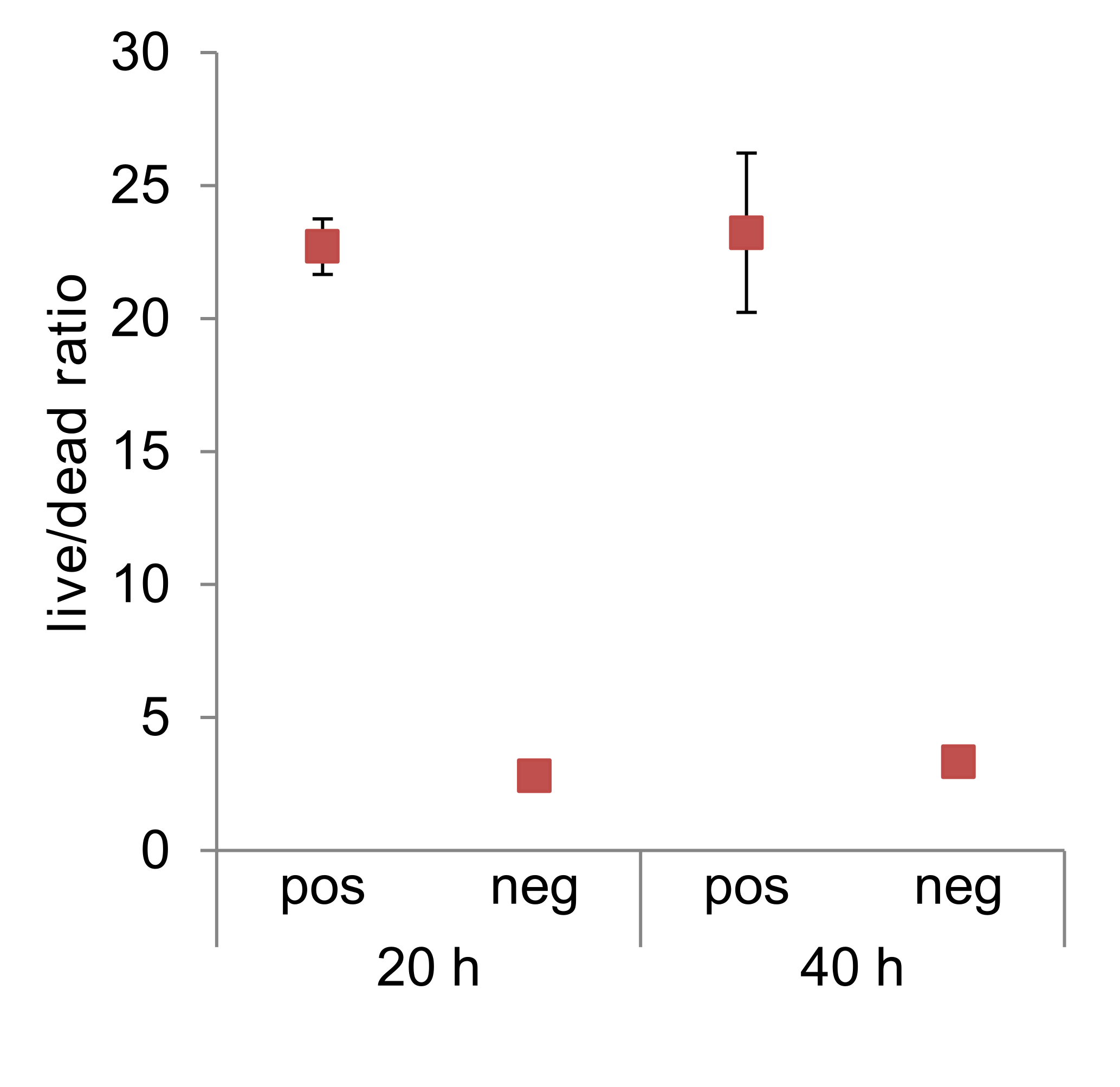

Supplement: S2 Fig — For the positive control, live/dead staining was performed with an untreated 20 h or 40 h old biofilm. The same biofilms were then killed with 70% 2-propanol for the negative control. (TIF) [file pone.0154086.s002.tif]

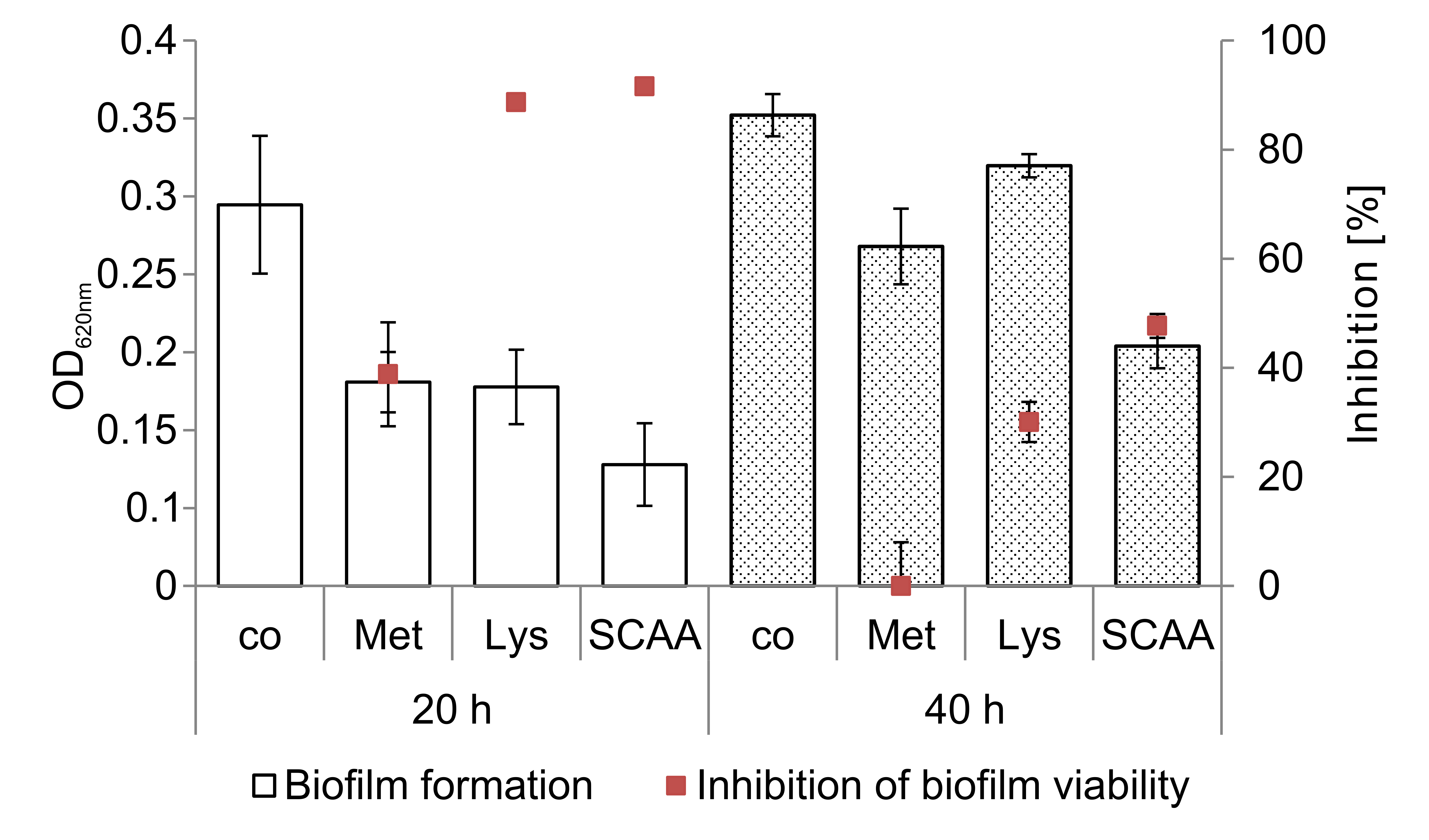

Supplement: S3 Fig — Maltose induced G. vaginalis biofilms were challenged with MET (0.1 mg/ml), LYS (0.5 mg/ml) and SCAA (1 mg/ml). Biofilm mass was determined by CV staining and is shown on the primary y-axis. Inhibition of biofilm viability [%] was measured via live/dead staining and is shown on the secondary y-axis. Mean and standard deviations from five replicates are shown. (TIF) [file pone.0154086.s003.tif]

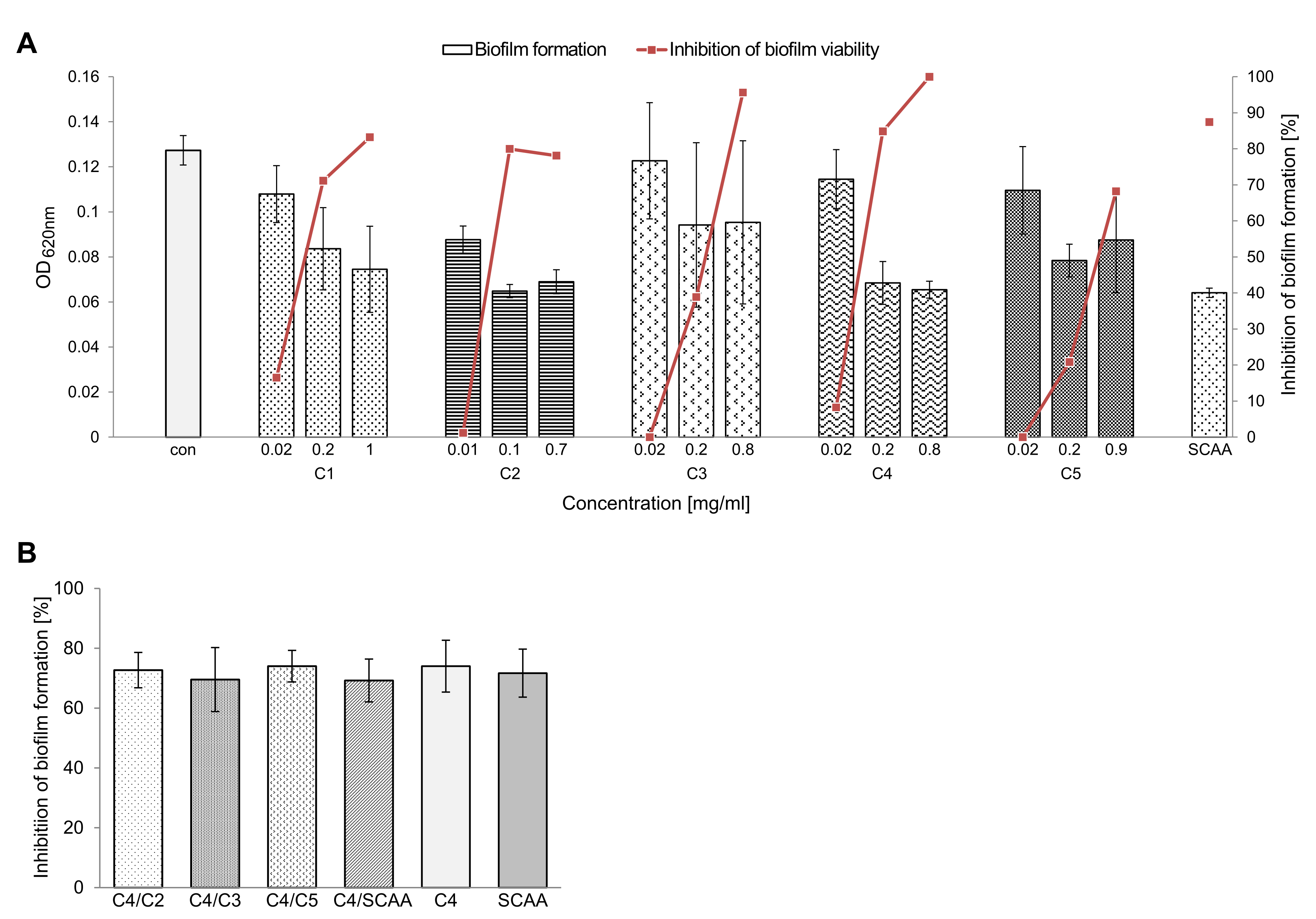

Supplement: S4 Fig — (A) C1, Cocoamidopropyl hydroxysultaine; C2, disodium cocoamphodiacetate; C3, sodium cocoamphopropionate; C4, sodium lauroamphoacetate; C5, cocoamidopropyl betaine (SCAA). (B) Effect of those tensides in different combinations with each other and with SCAA compared to C4 and SCAA alone. The primary y-axis shows biofilm mass determined by crystal violet staining, and the secondary y-axis shows inhibition of biofilm viability determined by live/dead staining. Mean and standard deviation from six replicate cultures are shown. (TIF) [file pone.0154086.s004.tif]
