# Supplementary material for: Screening of Compounds against Gardnerella vaginalis Biofilms
Source: PLoS One. 2016 Apr 25;11(4):e0154086. doi: 10.1371/journal.pone.0154086 (PMC4844189; doi:10.1371/journal.pone.0154086)
Supplement: S1 Table — (PDF) [file pone.0154086.s005.pdf]

	Abbreviation	Name	Company	Time	Concentration [mg/ml]	OD_{620nm}			Mean OD_{620nm} ± Std. dev.	Live/dead ratio			Mean live/dead ratio ± Std. dev.	Mean Viability Inhibition [%] ± Error
						Repl. 1	Repl. 2	Repl. 3		Repl. 1	Repl. 2	Repl. 3		
Antiseptics	CLC	Chlorocresol	Clariant GmbH	20 h	0	1,034	0,979	0,977	0,997 ± 0,032	10,881	10,889	11,241	11,004 ± 0,206	*
					0.1	0,421	0,417	0,376	0,405 ± 0,025	20,020	19,030	21,081	20,044 ± 1,026	0,000 ± 9,923
					0.5	0,000	0,003	0,004	0,002 ± 0,002	1,650	1,356	1,253	1,420 ± 0,206	87,095 ± 1,886
					1	0,001	0,000	0,002	0,001 ± 0,001	1,299	1,380	1,327	1,335 ± 0,041	87,866 ± 0,437
				40 h	0	1,230	1,221	1,030	1,160 ± 0,113	7,786	8,055	7,851	7,898 ± 0,140	*
					0.05	0,994	1,173	1,192	1,120 ± 0,109	8,554	7,827	8,520	8,300 ± 0,411	0,000 ± 5,525
					0.1	0,998	1,090	0,958	1,015 ± 0,068	8,319	8,583	7,862	8,255 ± 0,364	0,000 ± 4,975
					0.5	0,705	0,592	0,850	0,716 ± 0,129	3,257	3,312	3,794	3,455 ± 0,295	56,258 ± 3,820
	CPC	Cetylpyridinium chloride	Fagron GmbH & Co. KG	20 h	0	1,034	0,979	0,977	0,997 ± 0,032	10,881	10,889	11,241	11,004 ± 0,206	*
					2.5×10^{-4}	1,012	0,889	0,985	0,962 ± 0,065	11,814	11,175	10,992	11,327 ± 0,431	0,000 ± 4,366
					5×10^{-4}	0,715	0,714	0,767	0,732 ± 0,030	10,699	10,837	13,272	11,603 ± 1,447	0,000 ± 13,297
					0.001	0,000	0,003	0,007	0,003 ± 0,004	1,168	1,185	1,107	1,153 ± 0,041	89,519 ± 0,424
				40 h	0	1,071	1,136	0,961	1,056 ± 0,088	10,564	10,700	10,646	10,637 ± 0,069	*
					5×10^{-4}	0,502	0,542	0,598	0,547 ± 0,048	8,904	8,061	5,999	7,655 ± 1,495	28,036 ± 14,058
					0.001	0,617	0,537	0,571	0,575 ± 0,040	2,568	2,719	2,665	2,651 ± 0,076	75,081 ± 0,735
					0.005	0,606	0,600	0,474	0,560 ± 0,075	1,194	0,993	0,740	0,976 ± 0,228	90,829 ± 2,141
	PBI	Polyaminopropyl biguanide	Arch Biocides Ltd.	20 h	0	1,034	0,979	0,977	0,997 ± 0,032	10,881	10,889	11,241	11,004 ± 0,206	*
					0.01	0,002	0,003	0,033	0,013 ± 0,018	6,984	5,123	6,411	6,173 ± 0,953	43,902 ± 8,724
					0.05	0,003	0,003	0,003	0,003 ± 0,000	1,638	1,494	2,002	1,711 ± 0,262	84,449 ± 2,395
					0.1	0,000	0,012	0,000	0,004 ± 0,007	1,451	1,310	1,480	1,414 ± 0,091	87,151 ± 0,859
				40 h	0	1,500	1,495	1,251	1,415 ± 0,142	6,292	6,139	6,232	6,221 ± 0,077	*
					0.01	1,409	1,360	1,439	1,403 ± 0,040	5,092	6,181	5,798	5,691 ± 0,553	8,532 ± 8,955
					0.1	1,074	1,014	1,124	1,071 ± 0,055	7,035	6,413	7,262	6,903 ± 0,439	0,000 ± 7,195
					1	1,238	0,949	0,939	1,042 ± 0,170	15,124	14,647	14,017	14,596 ± 0,556	0,000 ± 9,392
Enzymes/ Peptides	OP1	OP-145	OctoPlus N.V.	20 h	0	0,931	0,892	0,973	0,932 ± 0,041	10,359	10,647	10,511	10,506 ± 0,144	*
					0.001	0,920	0,907	0,950	0,926 ± 0,022	12,256	10,674	10,183	11,038 ± 1,084	0,000 ± 10,414
					0.01	1,003	0,966	1,048	1,006 ± 0,041	13,447	12,836	11,727	12,670 ± 0,872	0,000 ± 8,464
					0.1	0,198	0,202	0,206	0,202 ± 0,004	1,237	1,371	1,431	1,346 ± 0,100	87,184 ± 0,964
				40 h	0	1,448	1,512	1,502	1,487 ± 0,034	9,184	9,259	9,399	9,281 ± 0,109	*
					0.001	1,436	1,468	1,543	1,482 ± 0,055	8,807	9,293	9,375	9,158 ± 0,307	1,320 ± 3,504
					0.01	1,346	1,468	1,364	1,393 ± 0,066	8,939	9,367	10,031	9,446 ± 0,550	0,000 ± 6,930
					0.1	1,526	1,573	1,553	1,551 ± 0,024	7,114	7,284	7,210	7,202 ± 0,085	22,396 ± 4,532
	LYS	Lysozyme	Biozym GmbH	20 h	0	0,935	0,980	0,985	0,967 ± 0,028	11,198	11,080	11,423	11,234 ± 0,174	*
					0.01	1,031	1,019	1,058	1,036 ± 0,020	10,955	10,899	11,885	11,246 ± 0,554	0,000 ± 5,170
					0.1	0,864	0,945	0,843	0,884 ± 0,054	9,162	9,013	7,144	8,440 ± 1,125	24,871 ± 10,078
					0.5	0,018	0,011	0,019	0,016 ± 0,004	1,445	1,265	1,462	1,390 ± 0,109	87,622 ± 0,991
				40 h	0	1,230	1,221	1,030	1,160 ± 0,113	7,786	8,055	7,851	7,898 ± 0,140	*
					0.5	1,349	1,571	1,388	1,436 ± 0,119	4,141	4,779	4,376	4,432 ± 0,322	43,881 ± 4,203
					2.5	0,809	1,117	1,095	1,007 ± 0,172	4,053	3,861	4,025	3,980 ± 0,103	49,607 ± 1,587
					5	0,925	0,767	1,018	0,903 ± 0,127	4,371	4,143	3,564	4,026 ± 0,416	49,021 ± 5,349
	PRO	Proteinase K	Fermentas GmbH	20 h	0	1,034	0,979	0,977	0,997 ± 0,032	10,881	10,889	11,241	11,004 ± 0,206	*
					0.004	0,712	0,515	0,089	0,439 ± 0,318	11,932	12,709	12,516	12,386 ± 0,405	0,000 ± 4,239
					0.02	0,015	0,080	0,030	0,042 ± 0,034	20,175	16,850	17,500	18,175 ± 1,762	0,000 ± 15,015
					0.1	0,006	0,008	0,053	0,022 ± 0,027	7,296	1,897	2,800	3,998 ± 2,892	63,669 ± 16,055
				40 h	0	1,500	1,495	1,251	1,415 ± 0,142	6,292	6,139	6,232	6,221 ± 0,077	*
					0.008	1,231	1,236	1,096	1,188 ± 0,079	6,958	6,962	7,916	7,279 ± 0,552	0,000 ± 8,990
					0.04	1,202	1,357	1,232	1,264 ± 0,082	9,177	7,823	8,378	8,459 ± 0,680	0,000 ± 11,062
					0.2	1,381	1,293	1,267	1,314 ± 0,060	7,599	7,805	7,789	7,731 ± 0,115	0,000 ± 2,402
Antibiotics	TOB	Tobramycin sulfate	Zhejiang Hisun Pharmaceutical Co. Ltd.	20 h	0	0,935	0,980	0,985	0,967 ± 0,028	11,198	11,080	11,423	11,234 ± 0,174	*
					0.01	0,717	0,735	0,752	0,735 ± 0,018	19,606	13,905	15,237	16,249 ± 2,982	0,000 ± 26,641
					0.1	0,006	0,003	0,005	0,005 ± 0,002	1,565	1,497	1,505	1,522 ± 0,037	86,448 ± 0,390
					1	0,001	0,004	0,001	0,002 ± 0,002	1,244	1,190	1,265	1,233 ± 0,039	89,026 ± 0,385
				40 h	0	1,230	1,221	1,030	1,160 ± 0,113	7,786	8,055	7,851	7,898 ± 0,140	*
					1	0,700	0,712	0,727	0,713 ± 0,014	7,629	7,864	9,322	8,271 ± 0,917	0,000 ± 11,764
					5	0,661	0,783	0,654	0,699 ± 0,073	10,054	9,933	10,040	10,009 ± 0,066	0,000 ± 2,405
					10	0,915	0,834	0,957	0,902 ± 0,063	11,900	10,925	8,942	10,589 ± 1,507	0,000 ± 19,232
	MET	Metronidazole	Biesterfeld Spezialchemie GmbH	20 h	0	0,931	0,892	0,973	0,932 ± 0,041	10,359	10,647	10,511	10,506 ± 0,144	*
					0.01	0,442	0,494	0,455	0,464 ± 0,027	17,187	14,204	14,968	15,453 ± 1,549	0,000 ± 14,884
					0.1	0,003	0,002	0,002	0,002 ± 0,001	1,896	1,833	1,756	1,828 ± 0,070	82,597 ± 0,707
					1	0,002	0,008	0,000	0,003 ± 0,004	1,737	1,666	1,827	1,743 ± 0,081	83,405 ± 0,802
				40 h	0	1,448	1,512	1,502	1,487 ± 0,034	9,298	9,259	9,289	9,282 ± 0,020	*
					0.01	1,338	1,332	1,266	1,312 ± 0,040	7,715	7,123	6,802	7,213 ± 0,463	22,287 ± 4,993
					0.1	0,776	0,892	0,730	0,799 ± 0,083	8,600	9,004	9,358	8,987 ± 0,379	3,179 ± 4,087
					1	0,610	0,656	0,783	0,683 ± 0,090	19,645	17,483	17,273	18,133 ± 1,313	0,000 ± 14,152
Tensides	LEC	Lecithin	Cargill GmbH & Co. KG	20 h	0	0,510	0,473	0,469	0,484 ± 0,023	10,881	10,889	11,241	11,004 ± 0,206	*
					12.5	0,067	0,092	0,120	0,093 ± 0,027	12,190	13,984	11,299	12,491 ± 1,368	0,000 ± 12,610
					25	0,177	0,111	0,162	0,150 ± 0,035	4,954	4,503	4,561	4,673 ± 0,245	49,658 ± 2,366
					50	0,329	0,406	0,620	0,452 ± 0,151	10,582	10,966	11,296	10,948 ± 0,357	0,000 ± 3,741
				40 h	0	1,071	1,136	0,961	1,056 ± 0,088	10,564	10,700	10,646	10,637 ± 0,069	*
					12.5	1,112	1,171	1,370	1,218 ± 0,135	17,508	16,980	18,373	17,620 ± 0,703	0,000 ± 6,696
					25	0,875	1,235	1,218	1,109 ± 0,203	16,770	13,130	12,647	14,182 ± 2,254	0,000 ± 21,206
					50	1,314	0,852	1,250	1,139 ± 0,250	14,916	13,798	14,403	14,372 ± 0,560	0,000 ± 5,332
	SCAA	Sodium cocoamphoacetate	C.H. Erbslöh KG	20 h	0	0,935	0,980	0,985	0,967 ± 0,028	11,198	11,080	11,423	11,234 ± 0,174	*
					0.25	0,010	0,004	0,002	0,005 ± 0,004	1,281	1,416	1,205	1,301 ± 0,107	88,423 ± 0,966
					0.5	0,000	0,003	0,004	0,002 ± 0,002	1,244	1,114	1,345	1,234 ± 0,115	89,012 ± 1,041
					1	0,005	0,001	0,027	0,011 ± 0,014	1,055	1,026	0,959	1,013 ± 0,049	90,981 ± 0,462
				40 h	0	1,230	1,221	1,030	1,160 ± 0,113	7,786	8,055	7,851	7,898 ± 0,140	*
					0.01	1,146	1,273	1,221	1,213 ± 0,064	6,902	7,213	7,033	7,049 ± 0,156	10,739 ± 2,533
					0.1	1,031	1,310	1,336	1,226 ± 0,169	7,668	11,999	8,143	9,270 ± 2,375	0,000 ± 30,147
					1	0,464	0,469	0,490	0,474 ± 0,014	3,231	3,083	3,032	3,115 ± 0,103	60,555 ± 1,482

* Viability inhibition values are dependent on the control

Table S1: Summary of all compounds including abbreviations, providers, tested concentrations and the effect on OD_{620nm} and live/dead ratio. Biofilm viability inhibition [%] was calculated based on the live/dead ratio.
